# P03.09. Development of an interprofessional model of collaborative care by doctors of chiropractic and medical doctors for older adults with low back pain

**DOI:** 10.1186/1472-6882-12-S1-P262

**Published:** 2012-06-12

**Authors:** C Goertz, S Salsbury, R Vining, A Andresen, M Hondras, M Jones, L Killinger, C Long, K Lyons, R Wallace

**Affiliations:** 1 Palmer Center for Chiropractic Research, Davenport, USA; 2 Genesis Quad Cities Family Medicine Residency Program, Davenport, USA; 3 Palmer College of Chiropractic, Davenport, USA; 4 Thomas Jefferson University, Philadelphia, USA; 5 The University of Iowa, Iowa City, USA

## Purpose

Although low back pain (LBP) is a common reason older adults seek treatment from either medical doctors (MD/DO) or doctors of chiropractic (DC), collaborative care between these providers is rarely reported. The purpose of our study is to develop a model for such collaborative care in LBP patients, based upon an existing integrative medicine model (Hsiao et al., 2006), focusing on four facets of interprofessional collaboration: attitudes, knowledge, referral, and integrative practice.

## Methods

An interdisciplinary committee composed of DCs, MDs, a pharmacist, biostatistician and health services researchers developed a collaborative care model for implementation within the context of a clinical trial. Family medicine physicians (n = 18) and chiropractic clinicians (n = 29) completed a survey, including the Integrative Medicine Questionnaire (IM-30). A pre-selected group of MD/DO residents and DC clinicians then completed five interprofessional education sessions (IPE) on the model and clinical trial, and participated in job shadowing experiences.

## Results

Median IM-30 scores measured pre-IPE indicated DC and MD/DO providers had comparable overall (61.1 vs 59.3) and subscale scores for openness to working with alternate paradigm practitioners (60.8 vs 55.0); readiness to refer patients to alternate paradigm practitioners (66.7 vs 66.7); and provision of patient-centered care (66.7 vs 75.0). DCs scored higher than MDs/DOs on subscales measuring willingness to learn from alternate paradigms (55.0 vs 30.0) and integrative medicine safety (80.0 vs 60.0). These data will be compared to Year 1 follow-up surveys currently underway. IPE fieldnotes documented collegial debate on the evidence-base supporting chiropractic and medical LBP treatments, clinical examination and imaging interpretation, and safety concerns.

## Conclusion

Early evaluation of our collaborative care model revealed similarities in DC and MD/DO attitudes towards working with alternate paradigm health practitioners on LBP care for older adults. Provider safety concerns and willingness to learn from alternate disciplines may require additional IPE training to overcome.

